# Supplementary material for: The NTR/prodrug revolution: Tools for controlling cell loss and regeneration
Source: eLife. 2026 Jun 5;15:RP110593. doi: 10.7554/eLife.110593 (PMC13241088; doi:10.7554/eLife.110593)
Supplement: Supplementary file 1. — This table lists the most highly cited publications employing the NTR/prodrug system for targeted cell ablation. Candidate studies were identified through a Web of Science search using the query ‘nitroreductase ablation’, and results were manually curated to include only those papers that directly used an NTR-expressing transgenic line or construct together with a prodrug to induce selective cell death. Entries are ranked by citation count at the time of data collection. For each study, the table reports the targeted cell type or tissue, the species and specific NTR transgenic line used, and the primary biological purpose addressed. The columns ad. (adult) and la. (larvae) indicate whether the transgenic system was used in adults, larvae, or both. The transgene shown in green corresponds to a widely used UAS line that drives Gal4-dependent expression of NTR1 and is included here to aid identification of experiments utilizing this common effector line. [file elife-110593-supp1.docx]

**Supplemental table 1**

| **Cell Type** | **Tissue** | **Transgenic line** | **ad.** | **la.** | **Purpose of Study** | **Ref.** |
| --- | --- | --- | --- | --- | --- | --- |
| Cardiomyocytes | Heart | *Tg(myl7:CFP-NTR)^s890^* |  | ✔ | Heart regeneration and functional recovery | ^1^ |
| Hepatocytes | Liver | *Tg(fabp10a:CFP-NTR)^s891^* |  | ✔ | Liver damage and regeneration | ^1^ |
| β-cells | Pancreas | *Tg(ins:NTR-mCherry)^JH4^* |  | ✔ | Regeneration and progenitor contribution | ^2^ |
| Gal4 expressing | Various | ***Tg(UAS-E1B:NTR-mCherry)^c264^*** |  | ✔ | bipartite approach to driving NTR | ^3^ |
| Rod cells | Eye | *Tg(zop:NTR-EGFP)^nt19^* | ✔ |  | Rod cell death activates muller glia | ^4^ |
| Rod cells | Eye | *Tg(rho:YFP-NTR)^gmc500^* |  | ✔ | Regeneration of rod cells | ^5^ |
| Microglia | Eye | *Tg(mpeg1.1:NTR-EYFP)^w202^* |  | ✔ | requires microglia for muller cell activation | ^5^ |
| podocytes | kidney | *Tg(nphs2:NTR-mCherry)* | ✔ | ✔ | glomerular pathogenesis and podocyte regeneration | ^6^ |
| motor neurons | CNS | *Tg(mnx1:GAL4)^s300t^,* ***Tg(UAS-E1B:NTR-mCherry)^c264^*** |  | ✔ | motor neurons (MN) regeneration | ^7^ |
| Mauthner cell | CNS | *Et(SCP1:Gal4ff^y264^, Tg(14xUAS-E1B:Ocu.Hbb2-NTR-TagRFPT-oPRE)^y268^* |  | ✔ | Confirming role of Mauthner cells | ^8^ |
| Glia | CNS | Tg(gfap:NTR-mCherry)^scz059^ and ^sc129^ |  | ✔ | showed radial glia are neural stem cells | ^9^ |
| UV cones | Eye | *Tg(opn1sw1:Gal4-VP16)^ua3016^;****Tg(UAS-E1B:NTR-mCherry)^c264^*** |  | ✔ | cone cell regeneration | ^10^ |
| cranial MN | CNS | *Tg(2xNRSE-isl1a-Mmu.Fos:KalTA4,5xUAS-E1b:GAP-YFP-2A-NTR_T41Q/N71S/F124T)^lmc004^* |  | ✔ | demo of new nfsB triple mutant | ^11^ |
| spinal MN | CNS | *Tg(2xNRSE-2xMnx1-Mmu.Fos:KalTA4,5xUAS-E1b:GAP-YFP-2A-NTR T41Q/N71S/F124T)^lmc008^* |  | ✔ | demo of new nfsB triple mutant | ^11^ |
| mature oligodendrocytes | CNS | *Tg(mbp:gal4-vp16);* ***Tg(UAS-E1B:NTR-mCherry)^c264^*** | ✔ | ✔ | demyelination in the spinal cord | ^12^ |
| oligodendrocyte lineage | CNS | *Tg(sox10:gal4-vp16);* ***Tg(UAS-E1B:NTR-mCherry)^c264^*** |  | ✔ | demyelination in the spinal cord | ^12^ |
| podocytes | kidney | Tg(nphs2:NTR-GFP)^zf526^ |  | ✔ | model to study podocyte regeneration | ^13^ |
| bipolar cells | Eye | Et(Gal4-VP16;UAS:eGFP)^xfz3^ and ^43^, **Tg(UAS-E1B:NTR-mCherry)c264** |  | ✔ | demo of enhancer trap and bipolar regen | ^14^ |
| satellite cells | muscle | *pax7a^gSAIzGFFD164A^;* ***Tg(UAS-E1B:NTR-mCherry)^c264^*** |  | ✔ | study of muscle wound repair | ^15^ |
| dopaminergic neurons | CNS | Tg(slc6a3:CFP-NTR)^ot1413^ |  | ✔ | locomotor function and regeneration | ^16^ |
| epidermal cells | Skin | Tg(krt4:NTR-hKikGR)^cy17^ | ✔ | ✔ | skin repair | ^17^ |
| retinal pigment epithelium | Eye | Tg(rpe65a:NTR-EGFP)^mw86^ | ✔ | ✔ | study or RPE regeneration | ^18^ |
| Cardiomyocytes | heart | Tg(myl7:mCherry-NTR)^s993^ |  | ✔ | study of myo and endocardial interactions | ^19^ |
| ostreoblasts medaka | skeleton | osx:CFP-NTR |  | ✔ | osteoblast function and regeneration | ^20^ |
| Müller glial cells *Xenopus laevis* | eye | Rho:GFP-Ntr |  | ✔ | Model generation of eye regen in frog | ^21^ |
| germ cells | gonads | Tg(dnd1:NTR-EGFP)^ihb116^ |  | ✔ | Sex determination in zebrafish | ^22^ |
| male germ cells | testes | Tg(AOS-eGFP:NTR)[Tg(Asp-eGFP:NTR; Odf-eGFP:NTR; Sam-eGFP:NTR)] |  | ✔ | creating male sterility in GMOs | ^23^ |
| female germ cells | ovary | g(ZP:NTR-EGFP) | ✔ |  | creating female sterility in GMOs | ^24^ |
| female germ cells | ovary | Tg(zp3:GAL4-VP16,myl7:CFP), **Tg(UAS-E1B:NTR-mCherry)^c264^** | ✔ |  | ovarian(and fertility) regeneration | ^25^ |
| β-cells | Pancreas | Tg(ins:FLAG-NTR,cryaa:mCherry)^s950^ |  | ✔ | mechanism of ablation study | ^26^ |
| pineal photoreceptors | CNS | Tg(gnat2:GAL4-VP16-EGFP)^nt24^, **Tg(UAS-E1B:NTR-mCherry)^JH17^** | ✔ |  | study into circadian rhythms | ^27^ |
| dopaminergic neurons | CNS | Tg[fuguth-1:gal4; **Tg(UAS-E1B:NTR-mCherry)^c264^** | ✔ | ✔ | screen for chemicals that protect dopaminergic neurons | ^28^ |

References

1. Curado S, Anderson RM, Jungblut B, Mumm J, Schroeter E, Stainier DYR. 2007. Conditional targeted cell ablation in zebrafish: a new tool for regeneration studies. Developmental Dynamics 236:1025–1035. DOI: https://doi.org/10.1002/dvdy.21100, PMID: 17326133
2. Pisharath H, Rhee JM, Swanson MA, Leach SD, Parsons MJ. 2007. Targeted ablation of beta cells in the embryonic zebrafish pancreas using E. coli nitroreductase. Mechanisms of Development 124:218–229. DOI: https://doi.org/10.1016/j.mod.2006.11.005, PMID: 17223324
3. Davison JM, Akitake CM, Goll MG, Rhee JM, Gosse N, Baier H, Halpern ME, Leach SD, Parsons MJ. 2007. Transactivation from Gal4-VP16 transgenic insertions for tissue-specific cell labeling and ablation in zebrafish. Developmental Biology 304:811–824. DOI: https://doi.org/10.1016/j.ydbio.2007.01.033, PMID: 17335798
4. Montgomery JE, Parsons MJ, Hyde DR. 2010. A novel model of retinal ablation demonstrates that the extent of rod cell death regulates the origin of the regenerated zebrafish rod photoreceptors. The Journal of Comparative Neurology 518:800–814. DOI: https://doi.org/10.1002/cne.22243, PMID: 20058308
5. White DT, Sengupta S, Saxena MT, Xu Q, Hanes J, Ding D, Ji H, Mumm JS. 2017. Immunomodulation-accelerated neuronal regeneration following selective rod photoreceptor cell ablation in the zebrafish retina. PNAS 114:E3719–E3728. DOI: https://doi.org/10.1073/pnas.1617721114, PMID: 28416692
6. Zhou W, Hildebrandt F. 2012. Inducible podocyte injury and proteinuria in transgenic zebrafish. Journal of the American Society of Nephrology 23:1039–1047. DOI: https://doi.org/10.1681/ASN.2011080776, PMID: 22440901
7. Ohnmacht J, Yang Y, Maurer GW, Barreiro-Iglesias A, Tsarouchas TM, Wehner D, Sieger D, Becker CG, Becker T. 2016. Spinal motor neurons are regenerated after mechanical lesion and genetic ablation in larval zebrafish. Development 143:1464–1474. DOI: https://doi.org/10.1242/dev.129155, PMID: 26965370
8. Tabor KM, Bergeron SA, Horstick EJ, Jordan DC, Aho V, Porkka-Heiskanen T, Haspel G, Burgess HA. 2014. Direct activation of the Mauthner cell by electric field pulses drives ultrarapid escape responses. Journal of Neurophysiology 112:834–844. DOI: https://doi.org/10.1152/jn.00228.2014, PMID: 24848468
9. Johnson K, Barragan J, Bashiruddin S, Smith CJ, Tyrrell C, Parsons MJ, Doris R, Kucenas S, Downes GB, Velez CM, Schneider C, Sakai C, Pathak N, Anderson K, Stein R, Devoto SH, Mumm JS, Barresi MJF. 2016. Gfap-positive radial glial cells are an essential progenitor population for later-born neurons and glia in the zebrafish spinal cord. Glia 64:1170–1189. DOI: https://doi.org/10.1002/glia.22990, PMID: 27100776
10. Fraser B, DuVal MG, Wang H, Allison WT. 2013. Regeneration of cone photoreceptors when cell ablation is primarily restricted to a particular cone subtype. PLOS ONE 8:e55410. DOI: https://doi.org/10.1371/journal. pone.0055410, PMID: 23383182
11. Mathias JR, Zhang Z, Saxena MT, Mumm JS. 2014. Enhanced cell-specific ablation in zebrafish using a triple mutant of Escherichia coli nitroreductase. Zebrafish 11:85–97. DOI: https://doi.org/10.1089/zeb.2013.0937, PMID: 24428354
12. Chung A-Y, Kim P-S, Kim S, Kim E, Kim D, Jeong I, Kim H-K, Ryu J-H, Kim C-H, Choi J, Seo J-H, Park H-C. 2013. Generation of demyelination models by targeted ablation of oligodendrocytes in the zebrafish CNS. Molecules and Cells 36:82–87. DOI: https://doi.org/10.1007/s10059-013-0087-9, PMID: 23807048
13. Huang J, McKee M, Huang HD, Xiang A, Davidson AJ, Lu HAJ. 2013. A zebrafish model of conditional targeted podocyte ablation and regeneration. Kidney International 83:1193–1200. DOI: https://doi.org/10.1038/ki.2013. 6, PMID: 23466998
14. Zhao XF, Ellingsen S, Fjose A. 2009. Labelling and targeted ablation of specific bipolar cell types in the zebrafish retina. BMC Neuroscience 10:107. DOI: https://doi.org/10.1186/1471-2202-10-107, PMID: 19712466
15. Pipalia TG, Koth J, Roy SD, Hammond CL, Kawakami K, Hughes SM. 2016. Cellular dynamics of regeneration reveals role of two distinct Pax7 stem cell populations in larval zebrafish muscle repair. Disease Models & Mechanisms 9:671–684. DOI: https://doi.org/10.1242/dmm.022251, PMID: 27149989
16. Godoy R, Noble S, Yoon K, Anisman H, Ekker M. 2015. Chemogenetic ablation of dopaminergic neurons leads to transient locomotor impairments in zebrafish larvae. Journal of Neurochemistry 135:249–260. DOI: https:// doi.org/10.1111/jnc.13214, PMID: 26118896
17. Chen CF, Chu CY, Chen TH, Lee SJ, Shen CN, Hsiao CD. 2011. Establishment of a transgenic zebrafish line for superficial skin ablation and functional validation of apoptosis modulators in vivo. PLOS ONE 6:e20654. DOI: https://doi.org/10.1371/journal.pone.0020654, PMID: 21655190
18. Hanovice NJ, Leach LL, Slater K, Gabriel AE, Romanovicz D, Shao E, Collery R, Burton EA, Lathrop KL, Link BA, Gross JM. 2019. Regeneration of the zebrafish retinal pigment epithelium after widespread genetic ablation. PLOS Genetics 15:e1007939. DOI: https://doi.org/10.1371/journal.pgen.1007939, PMID: 30695061
19. Palencia-Desai S, Rost MS, Schumacher JA, Ton QV, Craig MP, Baltrunaite K, Koenig AL, Wang J, Poss KD, Chi NC, Stainier DYR, Sumanas S. 2015. Myocardium and BMP signaling are required for endocardial differentiation. Development 142:2304–2315. DOI: https://doi.org/10.1242/dev.118687, PMID: 26092845
20. Willems B, Büttner A, Huysseune A, Renn J, Witten PE, Winkler C. 2012. Conditional ablation of osteoblasts in medaka. Developmental Biology 364:128–137. DOI: https://doi.org/10.1016/j.ydbio.2012.01.023, PMID: 22326228
21. Langhe R, Chesneau A, Colozza G, Hidalgo M, Ail D, Locker M, Perron M. 2017. Müller glial cell reactivation in Xenopus models of retinal degeneration. Glia 65:1333–1349. DOI: https://doi.org/10.1002/glia.23165, PMID: 28548249
22. Dai X, Jin X, Chen X, He J, Yin Z. 2015. Sufficient numbers of early germ cells are essential for female sex development in zebrafish. PLOS ONE 10:e0117824. DOI: https://doi.org/10.1371/journal.pone.0117824, PMID: 25679390
23. Hsu CC, Hou MF, Hong JR, Wu JL, Her GM. 2010. Inducible male infertility by targeted cell ablation in zebrafish testis. Marine Biotechnology 12:466–478. DOI: https://doi.org/10.1007/s10126-009-9248-4, PMID: 19936986
24. Hu S-Y, Lin P-Y, Liao C-H, Gong H-Y, Lin G-H, Kawakami K, Wu J-L. 2010. Nitroreductase-mediated gonadal dysgenesis for infertility control of genetically modified zebrafish. Marine Biotechnology 12:569–578. DOI: https://doi.org/10.1007/s10126-009-9244-8, PMID: 19941022
25. White YAR, Woods DC, Wood AW. 2011. A transgenic zebrafish model of targeted oocyte ablation and de novo oogenesis. Developmental Dynamics 240:1929–1937. DOI: https://doi.org/10.1002/dvdy.22695, PMID: 21761478
26. Kulkarni AA, ContehAM, SorrellCA, MirmiraA. 2018. An in vivo zebrafish model for interrogating ROS-mediated pancreatic beta-cell injury, response, and prevention. Oxidative Medicine and Cellular Longevity 2018:1324739. DOI: https://doi.org/10.1155/2018/1324739, PMID: 29785241
27. Li X, Montgomery J, Cheng W, Noh JH, Hyde DR, Li L. 2012. Pineal photoreceptor cells are required for maintaining the circadian rhythms of behavioral visual sensitivity in zebrafish. PLOS ONE 7:e40508. DOI: https://doi.org/10.1371/journal.pone.0040508, PMID: 22815753
28. Kim G-HJ, Mo H, Liu H, Wu Z, Chen S, Zheng J, Zhao X, Nucum D, Shortland J, Peng L, Elepano M, Tang B, Olson S, Paras N, Li H, Renslo AR, Arkin MR, Huang B, Lu B, Sirota M, et al. 2021. A zebrafish screen reveals Renin-angiotensin system inhibitors as neuroprotective via mitochondrial restoration in dopamine neurons. eLife 10:e69795. DOI: https://doi.org/10.7554/eLife.69795, PMID: 34550070
